# Supplementary material for: Anxiety and depression during pregnancy: Differential impact in cases complicated by preeclampsia and preterm premature rupture of membranes
Source: PLoS One. 2025 Apr 29;20(4):e0302114. doi: 10.1371/journal.pone.0302114 (PMC12040120; doi:10.1371/journal.pone.0302114)
Supplement: Attachment — Submitted filename: Comments_to_the_authors.docx [file pone.0302114.s001.docx]

Comments to the authors:

Title: Anxiety and depression during pregnancy: differential impact in cases complicated by preeclampsia and preterm premature rupture of membranes

The authors are trying to compare the three prospective cohort groups in terms of anxiety and depression during pregnancy. The objective of this study is to explore whether there is an increasing prevalence gradient of anxiety and depression among women with uncomplicated pregnancies, those admitted for PPROM, and those admitted for PE. The above objectives could be stated differently as it may be beneficial to compare the prevalence of the depression and anxiety among the three groups. Stated different, you could compare the prevalence of depression and anxiety between PPROM vs Control, PE vs Control, and perhaps PPROM vs PE.

Abstract:

The abstract is well written and there appears to be no significance difference between the groups: PPROM (27 vs. 24; p=0.049). Indeed, it is not clear if other covariate adjustments besides age have been done. It appears very little analysis result is presented in this section. Can you elaborate on the findings in the conclusion of the abstract?

Introduction

P4 l75-76: Rephrase to make this sound better. It is confusing in its current state.

P5l96-99: Break this to multiple sentences.

P5l100: remove “which”

Materials and design

Please include detail description of the different outcome variables.

L139: I believe the inclusion criteria had an upper band. Please describe this carefully. For example, women of childbearing ages 18yrs and older up to ….

Are the participants matched in terms of age at pregnancy or the number of prior births?

There may be several confounding variables that were overlooked.

The presentation of the study design is poor.

Study variables:

The two outcome variables: composite scores for the depression outcome and composite score for anxiety were reported. These were self-administered, meaning that there could be misinterpretation of the questions, social desirability bias, sampling bias.

It is also unclear how the if convenient samples were used.

It is unclear if any participant in study cohort A or B were allowed to choose to participate in the study or not. Elaborate on this.

Statistical analysis:

L192: “Qualitative variable” this should be replaced with categorical variable. It is impossible to characterized qualitative variable as frequency and percentages unless they are quantified.

The outcome variables are not normality distributed. It is unclear why the need to do the normality test using Shapiro Wilk’s test.

It is unclear how many times the measurement was taken for the pregnant women. Since this is a prospective cohort study, the assumption is that you follow the pregnant women until the event of interest occurs. Is there a baseline value? Were there no depression or anxiety at the onset? When did they develop depression and anxiety during the course of their pregnancy?

Please rewrite l195 -197. It is unclear if there is an inherent ordering of the group? If so, were you using a dichotomized outcome to do the trend test by Jonckheere–Terpstra?

Please clarify what dichotomization is been modelled and what model is been fitted.

It is unclear what the model/dichotomized outcome is been used to test the paired groups. Please clarify.

In the multivariate logistic model, again, the outcome variable is unclear. What is your dichotomization and why did you choose only gestational age and STAI-Trait score to be included in the model? Why were other variables collected not included in the model?

NOTE: The Chi-squared or One-way ANOVA methodology used to compare the groups does not appear to reveal what might be a critical point to consider. For example, a significant Anova results for the groups does not tell the reader which group differ. It only says that the at least one of the groups is different from the others.

Results:

L212-217 should be moved to study variable or material and design subsection

L217 should not have a period after PE as in “severe PE. Figure 1.”

When is baseline? What is the next follow-up time? If indeed there was a baseline, then it will be appropriate to model the change from baseline scores instead of what has been modeled.

What is the p-value p1 representing? Is it comparing the PPROM vs control or PPROM vs PE. It is not appropriate to do a three-way comparison and present p-values.

Suppose all p-values represents the comparison of PPROM vs Control group or PE vs Control group, then all significant p-values suggest you should adjust for those covariates in a multivariate model. This appears to have been omitted in the analysis.

The quantile regression results provide no explanation to what is going on. More covariates need to be explained in the model.

In fact, results are lacking and not sufficiently presented. Additionally, interpretation of the scanty tables presented are also lacking.

Summary and key results

It is unclear if the goal the authors set up is achieved in this instance. The summary of the key results was insufficient.

Again, the authors findings are unclear to say the least. If there are any findings, appropriate statistical methodology should be used to summarize the results. A discussion section should be used to explain what is going on. This is lacking in this study.

Research and clinical implications

The point is well noted, and your results and key points should be clearly pointed out in the result section and now supported in this section.

Study limitations and strengths

It does not appear you adjusted for confounders in the baseline covariates are state here:” Finally, we adjusted for confounders, 350 such as the baseline trait of anxiety and the gestational age at admission”.

Conclusion

Since the screening tools are not diagnostic tools, it is inappropriate to conclude using the results from this tool as a yard’s stick to classify prevalence of depression and anxiety among pregnant women ages 18 years and older.

Figure 1: This figure needs a title and a footnote description that makes it stand alone.

Figures 2 and 3: Needs a title and footnote description that makes it stand alone. The description should lead the reader to understand what they are looking at. For example, are the means/medians the same, smaller or larger? Is the variability similar or different?
