# Supplementary material for: Anxiety and depression during pregnancy: Differential impact in cases complicated by preeclampsia and preterm premature rupture of membranes
Source: PLoS One. 2025 Apr 29;20(4):e0302114. doi: 10.1371/journal.pone.0302114 (PMC12040120; doi:10.1371/journal.pone.0302114)
Supplement: Attachment — Submitted filename: Response to Reviewers.docx [file pone.0302114.s003.docx]

Barcelona, February 26th,2025

Dear Editor,

We welcome the opportunity to review our manuscript.

Find an itemized answer to each of the three points raised.

Best regards,

Francesc Figueras

**Journal Requirements:**

In the methods section please could you include the following information:

1. Details of the Sample Size Calculation / Power Calculation Used

We expected to recruit 50 women with preterm premature rupture of membranes (PPROM) and 50 with preeclampsia (PE) over the study period. An additional 100 uncomplicated pregnancies were intended to be included as a control group, resulting in a total planned sample size of 200 participants. To determine the adequacy of our sample size for detecting a trend in proportions across the study groups, we performed a power analysis using the linear-by-linear trend test. We assumed a significance level of 0.05 and three ordered exposure groups with expected proportions of 5%, 20%, and 30%. Using a trend test framework with equally spaced exposure levels, the estimated power for detecting a significant linear trend was 85.8%.

We added this paragraph to the Methods section in a revised version.

2. Additional Details on the Recruitment Methods Used

Women with complications requiring admission to the high-dependency unit of a referral hospital were recruited consecutively during the study period (December 2016–December 2018). Pregnant women without medical or obstetric complications, who attended routine antenatal care at the same institution during the study period, were recruited simultaneously alongside patients with complications.

We have accordingly reworded the paragraph in the Methods section where the recruitment is detailed.

3. Clarification on the Nature of the Study

Our study was a primary study. However, after the first submission of our manuscript, a study analyzing a smaller subgroup of patients (n=168) who had postpartum follow-up was conducted and published in the Spanish journal Atención Primaria (“Aten Primaria. 2024 Sep 25;57(3):103085. doi: 10.1016/j.aprim.2024.103085”). The data and analyses in the current manuscript were not included in that secondary study.
